# Comparison of methods for DE-CMR infarct size quantification - reproducibility among three core labs

**DOI:** 10.1186/1532-429X-15-S1-P180

**Published:** 2013-01-30

**Authors:** Igor Klem, Einar Heiberg, John D Grizzard, Lowie M Van Assche, Michele Parker, Hakan Arheden, Raymond J Kim

**Affiliations:** 1 Medicine, Duke University, Durham, NC, USA; 2 Cardiac MR group, Department of Clinical Physiology, Lund University Hospital, Lund, Sweden; 3 Radiology, Virginia Commonwealth University, Richmond, VA, USA

## Background

Infarct size measurements with delayed-enhancement cardiovascular magnetic resonance (DE-CMR) are being used as surrogate endpoints in acute myocardial infarction (AMI) trials comparing therapeutic strategies. Semiautomated techniques using signal intensity thresholding are thought to be more reproducible than manual planimetry to define AMI borders. For both methods, endo-/epicardial borders are determined by manual planimetry, which was not considered in prior reproducibility studies. Visual scoring of AMI size based on a standard 17-segment, 5-point score is faster and does not require planimetry of endo-/epicardial borders. We compared the reproducibility of visual scoring, manual planimetry and semiautomated techniques.

## Methods

Thirty patients with first AMI (58+/-11 years, 80% male), who underwent DE-CMR within 7 days after first elevated troponin test were enrolled. All scans were evaluated independently at each participating CMR core lab with at least three months temporal separation between analyses in the following manner: A) manual planimetry of the endo-/epicardial contours and infarct borders (MP), B) manual planimetry of endo-/epicardial contours, AMI size determined using a semiautomated technique with voxel weighting based on signal intensity, without user input (AUTO), C) same as B with user correction for no-reflow, artifact, etc. (AUTOcorrected), and D) visual scoring using a 17-segment, 5-point score (VISUAL). This comparison is based on a total of 30 measurements by three core-labs in four different manners for a total of 360 AMI size measurements.

Interobserver reproducibility was assessed using a) standard error of difference from mean (SED), and b) 1-intraclass-correlation coefficient (ICC). A SED<3 and 1-ICC<0.10 are considered excellent reproducibility.

## Results

The mean AMI sizes by MP as %LV were similar among 3 readers (20.5±11.02%, 20.02±11.59%, and 18.73±11.09%; p=NS). The results for the reproducibility are shown in the Table. The interobserver agreement between three readers for AMI size was excellent for all three techniques. Even with the use of a semiautomated technique without any user correction of AMI borders, there is variability in AMI size (AUTO: SED 2.90, 1-ICC 0.088), which is due to variability of the endo-/epicardial border planimetry. Variability of AUTO is improved after manual user correction of AMI borders (AUTOcorrected: SED 2.16, 1-ICC 0.043). Manual planimetry of endo-/epicardial and AMI borders as well as rapid visual scoring AMI size have a similar reproducibility to semiautomated techniques.

**Table 1:** Measures of Reproducibility

	Manual Planimetry	AUTO	AUTOcorrected	VISUAL
Standard error of difference (SED)	2.79	2.90	2.16	2.40

1-ICC	0.061	0.088	0.043	0.099

## Conclusions

The interobserver reproducibility of manual planimtery, semiautomated techniques, and visual scoring for AMI size quantification is similar and excellent. Variability of semiautomated techniques is due to planimetry of endo-/epicardial borders.

## Funding

NIH

